# Physical-Model-Checking to Detect Switching-Related Attacks in Power Systems

**DOI:** 10.3390/s18082478

**Published:** 2018-07-31

**Authors:** Mohamad El Hariri, Samy Faddel, Osama Mohammed

**Affiliations:** Energy Systems Research Laboratory, Department of Electrical and Computer Engineering, Florida International University, Miami, FL 33174, USA; sfadd002@fiu.edu (S.F.); mohammed@fiu.edu (O.M.)

**Keywords:** agent systems, cyber-physical security, decentralized control, intelligent systems

## Abstract

Recent public disclosures on attacks targeting the power industry showed that savvy attackers are now capable of occulting themselves from conventional rule-based network intrusion detection systems (IDS), bringing about serious threats. In order to leverage the work of rule-based IDS, this paper presents an artificially intelligent physical-model-checking intrusion detection framework capable of detecting tampered-with control commands from control centers of power grids. Unlike the work presented in the literature, the work in this paper utilizes artificial intelligence (AI) to learn the load flow characteristics of the power system and benefits from the fast responses of the AI to decode and understand contents of network packets. The output of the AI is processed through an expert system to verify that incoming control commands do not violate the physical system operational constraints and do not put the power system in an insecure state. The proposed content-aware IDS is tested in simulation on a 14-bus IEEE benchmark system. Experimental verification on a small power system, with an IEC 61850 network architecture is also carried out. The results showed the accuracy of the proposed framework in successfully detecting malicious and/or erroneous control commands.

## 1. Introduction

### 1.1. Motivation

Resilient and secure operation of the power grid relies on judicious cooperation between several cyber and physical entities. Cyber processes, for instance, read the physical states of the grid and interact with it by actuating physical devices. Therefore, communication signals could be feedback from sensory devices or control commands to actuating devices.

On several occasions, the literature has shown the ability of attackers to exploit vulnerabilities in the communication networks of electricity grids and tamper with control fields in network packets. For instance, [1] presented how a switching control command could be manipulated by an attacker to maliciously open circuit breakers causing blackouts. The work in [2] also showed how power could be interrupted by tampering with sensor measurements. Reference [3] showed that blackouts could be caused due to the sequential removal of substations or transmission lines by malicious acts. Similarly, Zhu et al. [4] discussed attack scenarios capable of causing cascaded failures in power systems.

Furthermore, recent public disclosures emphasized the brutality of control-related attacks on critical processes, such as the Stuxnet and the Crash Override malwares targeting industrial control systems and power plants [5,6]. In the Stuxnet malware, attackers targeted Programmable Logic Controllers (PLC)s by changing the control signals going to Variable Frequency Drives (VFD)s of motors. In a similar approach, the Crash Override malware targeted intelligent electronic devices by altering the switching commands sent to open and close circuit breakers.

Notwithstanding the fact that the aforementioned attacks targeted critical infrastructure, the gravity of these attacks is also accentuated by their ability to obscure themselves from conventional rule-based Intrusion Detection Systems (IDS)s. In such attacks, the modified control fields are re-encoded in the proper packet format before being transmitted on the network [1,7]. Rule based IDS rely on information in the header of network packets and compare them against standard stipulations or perform statistical analysis on network traffic to identify anomalous ones based on cyber rules. By that, conventional IDS disregard the actual data fields. Accordingly, there is a need for new innovative solutions that detect attacks that might disrupt of the operation of the power grid.

### 1.2. Related Work

Attacks on power control loops can be categorized as:

(1). False Data Injection Attacks (FDIA)s that target sensor or meter measurements. In these types of attacks, attackers attempt to feed back to the controller fake sensor measurements to alter its operation. For instance, a malware in [8] showed that injecting high current values into a substation’s network could cause controllers to issue unwanted trip signals, jeopardizing the reliability of the power system. There are extensive efforts on detecting and mitigating FDIAs. For instance, the authors in [9] presented an algorithm based on the linear Weighted Least Square Error (WLS) to detect bad or corrupted sensor measurements in digital substations. However, there are practical limitations on the WLS method, such as the latency. In [10], a false data injection attack detection mechanism, which based on identifying a set of candidate invariant microgrid parameters, was introduced. This method was designed specifically for DC microgrids. In [2], the authors focused on detecting fake sensor measurements in power systems and enhancing the reliability of power grid by forecasting the values of lost measurements, due to network congestion, based on historical trends. Similarly, there are plenty of other works that are focused on FDIAs, such as [11,12,13].
(2). Control-related attacks that target control commands going to actuators and field devices. There are few recent efforts that have been placed to detect control-related attacks in the energy sector that incorporates physical rules along with cyber rules. In [1], a semantic analysis framework, which integrates network IDS with power flow analysis was proposed to detect malicious control commands. To achieve acceptable detection latency, this technique requires adapting the power flow analysis algorithm, leading to a tradeoff between accuracy and latency, as the system expands. In [14], an anomaly detection algorithm, which is specific for detecting attacks on automatic generation control, is proposed. In the former, the control signal is executed on the physical system only if it is regarded as legitimate by the anomaly detection engine, otherwise, a signal from a model-based automatic generation control is utilized. This work relies on the assumption that the feed-back frequency and tie-line measurements are trusted and do not discuss their security requirements. In [15], faults are distinguished from cyber-attacks by following a mathematical formulation that incorporates PMU data, event status, and monitoring logs. Similarly, the work in [16] utilizes lookup tables for current measurements and circuit breakers statuses to compare current and previous states for attack detection. Both [15,16] require that data collection for the detection algorithms to be performed by a trusted entity, which is not always the case [17].

Therefore, there is a need for security systems that not only are capable of detecting anomalous network activity based on cyber rules, but also are aware of the content of network packets to be able to understand and assess their consequences on the physical grid. Since the goal of most attackers is to disrupt the operation of the power system, attackers have more incentive to directly alter control commands, rather than tamper with sensor measurements to affect the controllers’ actions. Accordingly, the focus of this paper will be on the detection of switching attacks on circuit breakers, which falls under the category of control related attacks.

### 1.3. Paper Contribution

This paper proposes a multi-agent security framework to detect and prevent cyber-attacks targeting circuit breakers in a power system. Unlike the work presented in the literature, the work in this paper utilizes artificial intelligence (AI) to learn the load flow characteristics of the power system and benefits from the fast responses of the AI to decode and understand the contents of network packets. The output of the AI is processed through an expert system to decide on whether an incoming control command contains malicious content or not.

The contributions of the paper are:

- While the work in the literature assesses network packets against mathematical models of the power system, to the best of the authors’ knowledge, this is the first effort to discuss the use of machine intelligence to develop a content-aware intrusion detection and prevention system that decodes and understands the physical meaning of the content of network packets.
- The use of AI reduces the online computational burden as compared to complex mathematical models and therefore, accelerates the attack detection and decision-making processes (in the μs range).
- Taking appropriate preventive action upon detecting malicious control commands and not only detecting intrusions.
- Finally, implementing the developed security multi-agent system on a hardware laboratory scale power system with an IEC 61850 communication architecture, taking into consideration the practical aspects that arise from the hardware implementation of the power system, agents as embedded microcontrollers, and communication network. The obtained results from the experimental setup proved the feasibility of the proposed security algorithm in real-time physical power systems.

The performance of the proposed framework was tested in simulation on a 14-bus IEEE benchmark system for 36 test cases covering all N-1 contingency scenarios. The results showed that all the malicious commands that will place the system in an insecure state, have been prevented from actuating the circuit breakers. The framework was also verified experimentally, where the security algorithm was compiled on embedded microntrollers that are interfaced with a 5-bus hardware power system testbed setup having an IEC 61850 communication infrastructure.

### 1.4. Paper Organization

The rest of the paper is organized as follows: Section 2 discusses the current standards used in power automation, describes the assumed attack models, and the details of the proposed model-checking approach. Section 3 presents and discusses the simulation results. Section 4 presents and discusses the agents’ development process, the hardware power system physical and network setup, and test results. Section 5 concludes the paper and proposes future work.

## 2. The Artificially Intelligent Physical-Model Checking Approach

**Figure 1:**
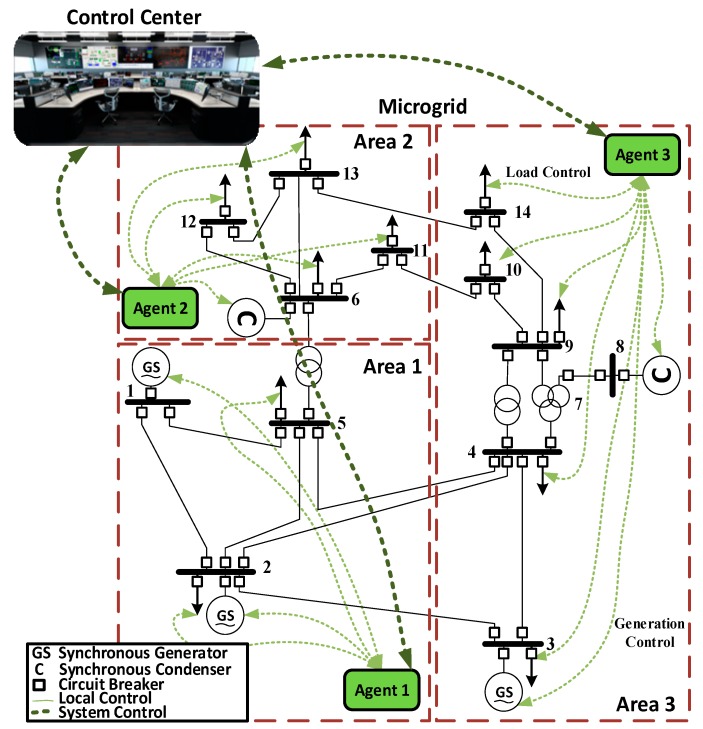
14-Bus IEEE benchmark system with decentralized and hierarchical control.

The proposed multi-agent security framework is shown in Figure 1. In this work, the power system is sectionalized into several zones. In each zone, an agent is responsible for: (1) local control and (2) security actions.

In terms of local control, each agent is interfaced with sensors, actuators, and field devices within its zone. Based on the feedback that it gets from the sensors, agents control the actuators and the field devices. The agents communicate among each other to achieve the global objective of stable and reliable operation of the power system. The agents also act as mediators between control centers and field devices/processes. This will allow control centers to poll information/data from sensors to monitor the entire system and to send control actions to actuators and field devices. Such a control architecture is referred to as decentralized control. The standard industrial communication protocols that allow the adoption of the decentralized control are discussed in Section 2.1.

Unlike centralized control, the decentralized multi-agent framework in this paper contributes to the security of the power system by avoiding single points of failure. In centralized control, all the sensors and field devices communicate with a single server, which from a security view-point is a bottle-neck and single point of failure. However, in decentralized control, even if one of the agents failed, the system will not entirely collapse. In addition to that, by processing data locally and performing local control actions, the amount of data to be transferred to the control center and the communication bandwidth will be reduced. The required processing power on control centers will be less and the system reliability will be improved.

In terms of the security actions, the goal of the proposed algorithm is to add an additional security layer to the operation of the power system by detecting malicious actions, which might occur on switching commands traveling the communication network. Switching commands that come from the system operator can be tampered with. The agents in this work are capable of assessing the physical consequences of these switching commands before they are executed, to ensure reliable operation of the power system and that the voltage levels and line loadings do not violate the safe operational limits set by standards [18,19]. The agents’ ability to assess the physical consequences is coming from intensive training of its neural network, by performing all the possible N-1 contingency analysis and all possible switching attack combinations on the loads and generators, and feeding all this data to the neural network. The proposed security algorithm is discussed in detail in Section 2.2.

### 2.1. Current Standards and Associated Cyber Threats

The two most used protocols for system automation and control in the power industry are the Distributed Network Protocol (DNP3.0) for Supervisory Control and Data Acquisition (SCADA) systems and IEC 61850 Manufacturing Message Service (MMS), Generic Object Oriented Substation Event (GOOSE), and Sampled Measured Values (SMV) messages in more recent systems [1,14]. Although these protocols enabled decentralized, robust, and more accurate power system control, they brought alongside some vulnerabilities in terms of cyber security. Each of the aforementioned protocol suits has its own vulnerabilities that were previously exploited to launch successful attacks on power grids. For instance, Lin et al. [1] presented a successful data manipulation attack on a DNP3 packet which has 4 control relay objects to operate 4 circuit breakers in a substation. A GOOSE poisoning attack was also presented in [7] to generate malicious circuit breaker switching commands as GOOSE messages. As mentioned previously, these attacks remained obscure form the network IDS since the attackers established fake data as legitimate network packets but with malicious content.

A major facilitator of such attacks is that power system communication networks need to accommodate the real-time operation of the grid. Therefore, strict time delay requirements are imposed on the exchange of communication signals. Since current microcontrollers and Intelligent Electronic Devices (IEDs) have low processing power, such industrial control networks are left unencrypted, and sometimes, without authentication. In fact, a study conducted in [20] shows that even the latest processor technologies cannot meet the 4 ms end-to-end time delay requirement set forth by the IEC 61850 standard stipulations on GOOSE messages.

Since this work targets the detection of control-related attacks, the publisher/subscriber GOOSE messaging protocol is selected for controlling the statuses of circuit breakers in the studied system. As will be shown later in the paper, the latency of the proposed detection algorithm falls within the 4 ms time delay set for GOOSE messaging.

#### Attack Model

In order to understand the threat models assumed in this paper for DNP3.0 and GOOSE switching commands, we differentiate between two types of switching commands, as in [1]:

1. Automatic Switching Commands: These are commands exchanged between IEDs/Agents to clear short-circuit faults, and they are usually exchanged over a Local Area Network (LAN). Typically, these messages are either DNP3.0 switching commands or GOOSE commands.
2. Manual Switching Commands: These are commands sent by the system operator in the control center over a Wide Area Network (WAN). These messages could be either DNP3.0 or Routable GOOSE (R-GOOSE) messages, as defined in IEC TR 61850-90-5 for Routable GOOSE over WAN.

**Figure 2:**
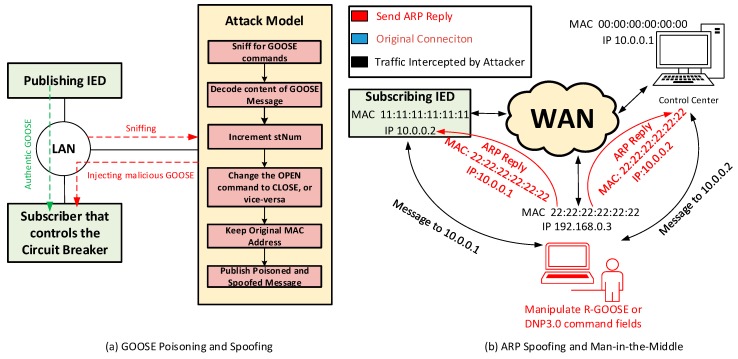
(**a**) Goose Poisoning and Spoofing Procedure; (**b**) ARP Poisoning and R-GOOSE and DNP3.0 Man-in-the-Middle Attack.

As reported in Table 1, GOOSE messages are Layer 2 messages of the Open System Interconnect (OSI), which are exchanged over a LAN. The OSI model divides a network into seven abstraction layers with the goal of providing interoperability to communication systems. In this work, we assume that an attacker is able to perform a GOOSE Spoofing and Poisoning attack. First, the attacker sniffs the network for GOOSE messages. Since these messages are unencrypted, the attacker could decode the content of the GOOSE message and modify the data fields (i.e., change the OPEN command to CLOSE, or vice-versa). Here, it is important to understand that GOOSE messages are event-driven, and each message is associated with an incremental counter, called status number (stNum). For example, the IED starts by sending a GOOSE message with stNum = 1. If a fault happens, the IED senses this fault and issues a new GOOSE commands with stNum = 2, to open the circuit breaker and clear the fault. Knowing that, the attacker then publishes the poisoned GOOSE message with a new incremented stNum and a spoofed MAC address. That is, the attacker uses the MAC address of the original sender. This process is depicted in Figure 2a.

Table 1 also shows that R-GOOSE and DNP3.0 are Layer 3 and Layer 4 messages, respectively. This means that they are exchanged over an IP network. Accordingly, they are susceptible to Address Resolution Protocol (ARP) Poisoning and Man-in-the-Middle attacks. ARP is a communication protocol used to convert IP addresses into MAC addresses [21]. As shown in Figure 2b, the attacker sends an ARP Reply to install a fake IP address and MAC address mapping to other hosts on the network. Therefore, the IP address of the attacker is, now, associated with an incorrect MAC address. This allows the attacker to intercept the messages exchanged between the control center and the subscriber IED. The attacker can, then, perform a Man-in-the-Middle attack and manipulate the data fields in the R-GOOSE or DNP3.0 packets.

Finally, although most industrial communication networks are not open to the public internet, we assume that they can still be penetrated through corporate networks or personal devices of the employees with techniques such as password cracking, backdoors, and malwares among others [1].

### 2.2. The Proposed Security Algorithm

**Figure 3:**
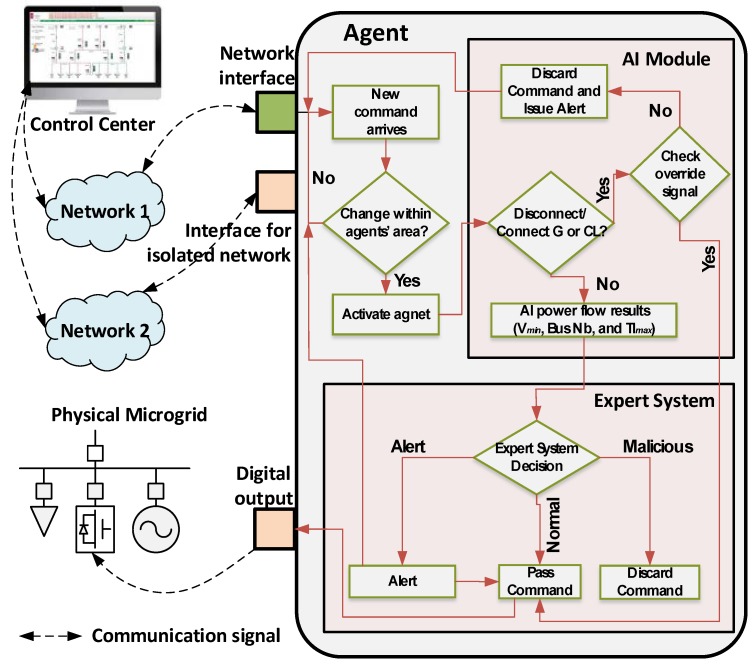
Agent operation and the physical-model-checking approach.

A block diagram of the security module of each agent and its network interfaces is shown in Figure 3. As can be seen in Figure 3, the security functionality in each agent is divided into two layers: an AI module and an Expert System Module.

#### 2.2.1. The AI Module

In the first layer, the agent is continuously listening to incoming control commands from the control center through its Ethernet network interface. Once a command is received, the agent decodes the content of the network packets and checks if the requested change is within its area or not. The agent gets activated only if the change is within its area. Once the agent is activated, the AI module will check if the command is to disconnect a generation unit or a critical load. This command will be further processed only if the agent sees an override signal from the system operator over its isolated network interface. For all other commands, the AI module will pass the commands through a trained neural network that will solve the power flow problem for the system. The AI module will output the minimum voltage in per unit, the bus number on which this minimum voltage is anticipated, and the maximum transmission line overloading. The neural network (NN) in this work is a feed forward NN trained using the back-propagation algorithm. The mathematical process guarding the operation of the AI module are explained below.

The NN utilized in this model is a three layer one composed of an input layer, a hidden layer, and an output layer. Let $X1[I+1]=\{x1_{1},x1_{2},\ldots ,x1_{i},1\}$ be the input coming to the NN, where i∈{1,I},I is the dimension of the input, and $x1_{I+1}=1$ is the bias for the input layer. In the input layer, the inputs are multiplied by the weights ($w1_{h,i}$) to get the vector $N1[H]=\{n1_{1},n1_{2},\ldots ,x1_{h}\}$. H is the dimension of the hidden layer and h∈{1,H}. The elements of N1 are calculated in accordance to (1):

(1) $$n1_{h}=\sum_{i\;=\;1}^{I+1}x1_{i}\times w1_{h,i}$$

Next, each element in N1 will be processed through a neuron in the hidden layer, which will result in $X2[H+1]=\{x2_{1},x2_{2},\ldots ,x1_{h},1\}$. The elements in X2 are calculated according to (2) and (3):

(2) $$x2_{h}=\frac{2}{1+e^{-n2_{h}}}-1$$

(3) $$x2_{H+1}=1$$

$x2_{H+1}$ is the bias of the hidden layer. The equation in (2) is considered as the activation function for the hidden layer neurons, which represents a sigmoid function.

Finally, X2 will be processed by the output layer to get the output vector $O[K]=\{o_{1},o_{2},\ldots ,o_{k}\}$, where K is the dimension of the output and k∈{1,K}. The elements in O are calculated according to (4):

(4) $$o_{k}=\frac{2}{1+e^{-\sum_{h=1}^{H+!}x2_{h}\times w2_{h,k}}}-1$$

$w2_{h,k}$ are weights between the hidden and output layer. The sigmoid function is also used as the activation function for the neurons in the output layer as in (4).

The output vector is then interpreted to get the minimum voltage, the bus number at which this voltage occurred, and the maximum transmission line loading.

#### 2.2.2. The Expert System Module

This module consists of a fuzzy inference system. The outputs of the AI module, are passed to the fuzzy inference system for further processing. These are the minimum bus voltage recorded in the power system, the number of the bus at which that minimum voltage occurred, and the value of the maximum transmission line overloading. It is to be noted here that the expert system module does not account for over-voltage cases. This is due to the fact that such cases will be the result of disconnecting multiple loads from the power system; however, the proposed security algorithm will automatically counteract such incidents.

The value of the bus voltage, which is passed to the expert system module, is fuzzified using four membership functions. These membership functions are designed to reflect the behavior of the power system according to the recommendations of the ANSI C84.1-2006 standard, from the voltage point of view [18]. The membership functions that correspond to the voltage are:

- Very Low (VL), repressing severe under voltage and Very High (VH), representing severe over voltage.
- High (H), representing normal condition and Low (L), representing mild undervoltage. For the latter case, a corrective action is necessary, such as reactive voltage support.

In this work, it is assumed that different priorities are assigned to the power system buses by the system operator. That is, high priorities will be assigned to the buses where main generator units or critical loads are connected, whereas low priorities will be assigned to busses with normal loads that can be shed. Due to the limited capacity in the generators adopted in this model, all the buses with generators were considered as critical buses (buses 1, 2, 3, 6, and 8), because the disconnection of any of the generators can result in cascaded failures in the whole system, which will result in a partial or full blackout. Also, generators are expensive equipment and are hard to replace within a reasonable time during emergencies. The study by Assante, which was conducted in 2007, showed how a massive diesel generator could be physically and permanently broken with only digital commands [4]. In fact, manipulating digital commands actually occurred in real life in the Crash Override malware. The behaviour of the power system in response to the Crash Override malware that targeted the Ukrainian power grid was considered as an example in assigning the critical busses in the test bench system presented in this paper [5]. In the aforementioned malware, the attackers targeted switching commands from the SCADA system, which controlled the status of circuit breakers. They were able to de-energize some of the substations resulting in blacking out a portion of the Ukrainian capital equivalent to a fifth of its total power capacity. As reported in [5]: “The command sequence polls the target device for the appropriate addresses. Once it is at the subset of known addresses, it can then toggle the value. The command then begins an infinite loop and continues to set addresses to this value effectively opening closed breakers. If a system operator tries to issue a close command on their HMI the sequence loop will continue to re-open the breaker. This loop maintaining open breakers will effectively de-energize the substation line(s) preventing system operators from managing the breakers and re-energize the line(s).”

We assigned the loads at buses 2, 3, 4, and 9 to be critical loads. The bus number is therefore considered as another second input to the fuzzy system. Finally, the fuzzy system takes the maximum transmission loading (TLL_max_) as its third input. The membership functions for this input are divided according to the rating procedures, that can be found in [19], to normal loading (represented by N), allowable overloading (represented by LTE), and unallowable overloading (represented by STE). The acronyms are in accordance with those is [19].

In this work, the trapezoidal membership functions shown in relation (5) were considered:

(5) $$f(x)=\left\{ \begin{array}{ll} 0, & \left(x<a)\cup (x>d\right)\\ \frac{x-a}{b-a}, & a\leq x\leq b\\ 1, & b\leq x\leq c\\ \frac{d-x}{c-d}, & c\leq x\leq d \end{array}\right.$$

The boundaries {a,b,c,d} for each input level is defined in Table 2. 

As for the bus numbers input, impulse membership function are defined as in (6):

(6) $$f(x)=\left\{ \begin{array}{ll} 1, & x\in \{1,N\}\\ 0, & otherwise \end{array}\right.$$

where N is the number of busses in the system and $x\in Z^{+*}$.

The output of the expert system is defuzzified based on weighted sum the Sugeno technique following Equation (7):

(7) $$output=\frac{\sum w_{i}x_{i}}{\sum w_{i}}$$

Since this is a Sugeno-like fuzzy system, the output is a number, which value is interpreted to be one of the following three cases: normal, alert, or malicious. In the normal case, the control commands are executed as they come, whereas in the malicious case, the commands are blocked, since they are suspected to put the power system in an insecure state. Finally, in the alert case, the control commands are executed, but an alert is passed to the system operator over the out-of-band channel to take corrective actions, if necessary. The controller makes its decision according to the following four fuzzy rules:

1. For the cases in which the control commands will lead the power system to have extreme over-voltage (represented by VH) or extreme under-voltage (represented by VL) on any of its busses, or extreme overloading of any of its transmission lines (represented by STE), the control command will be considered malicious by the fuzzy inference system. This is because, under no reasonable circumstance, will the system operator perform circuit breakers switching actions that will put the power system in an insecure state, which might be the cause of cascaded blackouts.
2. For the cases in which the control commands will lead the power system to have low voltages (represented by L) on one of the busses that has a main generator and/or a critical load connected to it, the fuzzy inference system will consider command will also consider the command as malicious. This is because such critical buses must maintain good voltage conditions at all times.
3. For the cases in which the voltage ends up to be low (represented by L) on the buses, which are not accounted for in the second rule (Rule 2), or when the transmission line loading condition is expected to be LTE, the control commands will be passed. However, the system operator will receive an alert signal over the out-of-band channel, in order to see if further actions are necessary.
4. For the remaining cases, in which the incoming control commands do not place the power system in an insecure state, the fuzzy inference system will pass the control commands without any issue.

Based on the previous rules, and following the standards for voltage rating and line loading [19,20], respectively, the voltage at the different busses should be maintained within acceptable limits and the line loading should be maintained within certain values to avoid overheating. Accordingly, the expert system was designed to ensure that any malicious action, which is anticipated to put any part of the system outside the acceptable standard limits, whether it is voltage or line loading or loss of generation or critical loads, will not be processed.

It is important to note here that the system operator will need to perform some tasks, such as maintenance tasks, which require the temporary disconnection of transmission lines or shutdown of generators. For that purpose, the system operator has the ability to communicate with all the agents over an encrypted and out-of-band communication channel and send a signal to override the decision of the agents.

## 3. Simulation Results

First, the proposed algorithm was verified in simulation on the 14-bus IEEE benchmark system, which is shown in Figure 1. The parameters of the system are given in Appendix A. The system was divided into three zones, in such a way that each zone has at least one generation unit and one load. Specifications of the system are found in detail in [22].

As mentioned earlier, the AI module of the first layer of the agent is trained to learn the characteristic of the system. This is done according to the following procedure:

- *Generating the Training and Test Target Data Sets*: First, in this work, we assume *N*-1 contingency cases (i.e., disconnecting the transmission lines, one at a time) and the disconnection of the generators and the loads. For the simulated 14-bus system, this gives us 36 cases plus the normal case, where all the circuit breakers are closed. For each case, the power flow problem was solved and the real power (*P*), reactive power (*Q*), and bus voltage (*V*) at the different busses, and transmission line loading (*TLL*) results were recorded as follows:
  $$\left[\begin{array}{cccccccccccc} P1Bus1 & \ldots & P1BusN & Q1Bus1 & \ldots & Q1BusN & V1Bus1 & \ldots & V1BusN & TLL11 & \ldots & TLL1K\\ P2Bus1 & \ldots & P2BusN & Q2Bus1 & \ldots & Q2BusN & V2Bus1 & \ldots & V2BusN & TLL21 & \ldots & TLL2K\\ \vdots & \vdots & \vdots & \vdots & \vdots & \vdots & \vdots & \vdots & \vdots & \vdots & \vdots & \vdots \\ \vdots & \vdots & \vdots & \vdots & \vdots & \vdots & \vdots & \vdots & \vdots & \vdots & \vdots & \vdots \\ P37Bus1 & \ldots & P37BusN & Q37Bus1 & \ldots & Q37BusN & V37Bus1 & \ldots & V37BusN & TLL371 & \ldots & TLL37K \end{array}\right]$$
  where, [*P_i_Bus*1 … *P_i_BusN*], [*Q_i_Bus*1 … *Q_i_BusN*], and [*V_i_Bus*1 … *V_i_BusN*] are the real power, reactive power, and voltage of the system busses, respectively, and i∈{1,37};N=14. [*TLL_i_*1 … *TLL_i_K*] are the transmission lines loading, and K=20.
- *Generating the Training and Test Input Data Sets*: Second, in this case study, the switching commands corresponding to each contingency were mapped to a 6-bit binary code, as shown in the matrix below. For instance, the code 000010 will be utilized to represent a control signal to actuate circuit breakers connecting bus 1 to bus 2.
  $$\left[\begin{array}{cccccc} 0 & 0 & 0 & 0 & 0 & 1\\ 0 & 0 & 0 & 0 & 1 & 0\\ 0 & 0 & 0 & 0 & 1 & 1\\ \vdots & \vdots & \vdots & \vdots & \vdots & \vdots \\ 1 & 0 & 0 & 1 & 0 & 1 \end{array}\right]---->\left[\begin{array}{c} Case1\\ Case2\\ Case3\\ \vdots \\ Case37 \end{array}\right]$$
- *Training the Neural Network*: The neural network of the agent was trained according to the Back Propagation algorithm to accurately predict the system response in terms of the real power, reactive power, and voltage of the buses and the transmission line loading.

**Figure 4:**
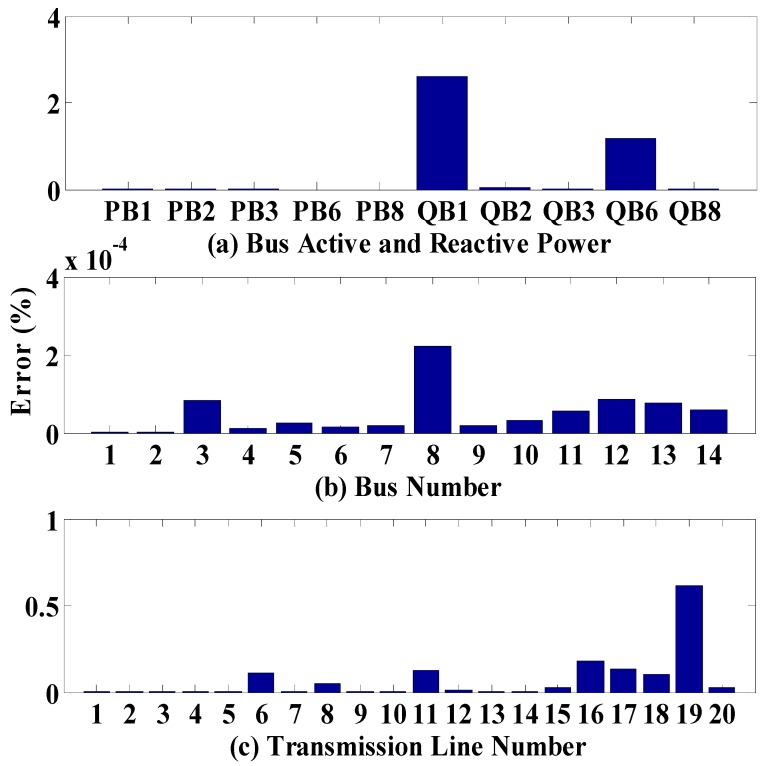
Mean Squared Error: (**a**) Active and Reactive Power on Generator Busses; (**b**) Bus Voltages; (**c**) Transmission Line Loading.

The accuracy of the neural network is shown in Figure 4. The maximum errors recorded were 2.6% for the active and reactive power, 10^−4^ for bus voltages, and 0.614% for transmission line loading.

Simulation of the proposed framework were carried out in two different scenarios and the results are tabulated in Table 3. In the first scenario, the response of the agents is assessed for each contingency case separately. That is, after each command, the system is reverted to normal case before executing the next command. The results for the first scenario are as follows:

- Cases 21–25 and 29–31 correspond to the disconnection of either a generation unit or a critical load from the system. Since the override signal was set to zero throughout the experiment, these commands were regarded as malicious and, therefore, were not passed to the circuit breakers.
- Cases 9, 11, 12, 15, 18–20, 27, 28, and 33–36 resulted in alert situation, where the commands were passed but an alert was issued to the system operator. It was noticed that there were no severe bus voltage deviations in these cases from the allowable limits. In fact, in all the cases, the minimum bus voltage was 1.02 p.u. Also, the maximum recorded transmission line loading was 129%, which falls into the LTE state of Table 2.
- Cases 1–4, 7, 8, 10, and 14 violated the physical operation constraints of the power system, and thus, were regarded as malicious by the agents.
- The rest of the cases were regarded as normal. It is worth noting that in these cases, commands to disconnect non-critical loads, such as cases 27 and 28, were passed. Although these commands might be malicious or erroneous, they were passed by the multi-agent system since they did not put the power system in a contingency state and the power system maintained its stability. Therefore, the multi-agent system was successful in satisfying its purpose by ensuring that only signals that do not violate the stable operational limits of the power system will be passed.

Therefore, out of all simulated cases, the multi-agent security framework allowed the passage of a command that lead to disconnection of a non-critical load 2 times. This is equivalent to 5.56% of the simulated cases. To address this issue, each agent generates periodic log reports and sends them to the system operator over its out-of-band network interface. This allows the system operator to get feedback about the state of the circuit breakers and utilizes this feedback to take corrective actions, if required.

It is worth noting that in the simulation case study, the dynamic analysis against time is not considered, since the training and the response of the agents in this paper are based on the steady-state contingency analysis. Additionally, each case in Table 2 represents an attack on a different circuit breaker, thus each case has a different power flow and plotting all the 36 cases over time does not show indicative information and is infeasible in this manuscript. For example, the normal operation of the system has a certain voltage, power, and current distribution and during the attack, one of these (either the voltage or the line current) will slightly or drastically deviate and needs to be compared with the standard ratings. Based on that, one of the agents will either pass the switching command, block it, or alert the system operator. Therefore, following Table 2 can give a good indication of what has changed, compared to the normal case, and the response of the agents.

**Figure 5:**
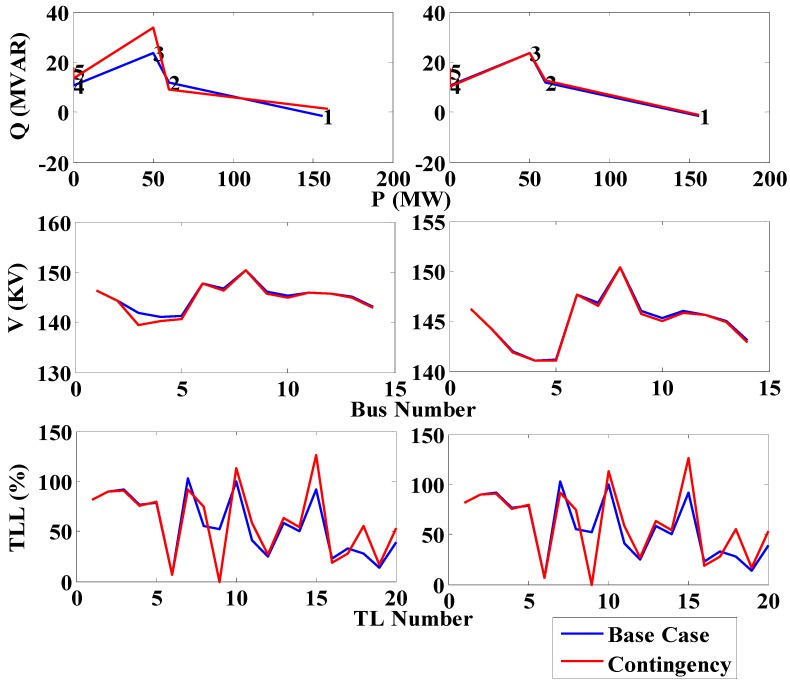
(**Left Column**) Comparison of *P* and *Q*, *V*, and *TLL* of a Malicious Case to the Base Case; (**Right Column**) Comparison of *P* and *Q*, *V*, and *TLL* of an Alert Case to the Base Case.

Figure 5 shows a sample of report completed based on the log reported by the agents. The left column compares *P*, *Q*, *V*, and *TLL* of a malicious case (Case 3) to the normal case. The right column compares the same for an alert case (Case 9) to the normal case. The reports show that for Case 3, if the control commands were to be executed, the power system would have gone into an insecure state. On the other hand, the control commands would not have a significant impact on the operation of the power system for Case 9.

Therefore, these reports could be considered very useful visualization tools that would assist in future plans and lessons learned.

## 4. Hardware Setup and Experimental Results

### 4.1. Description of the Hardware Setup

**Figure 6:**
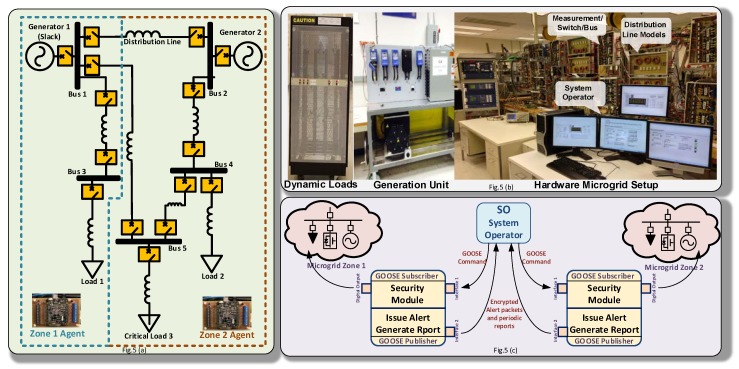
(**a**) One-line diagram of the 5-bus hardware microgrid; (**b**) Actual laboratory setup; (**c**) Network topology of agents.

The performance of the proposed security framework was tested on the 5-bus power system shown in Figure 6a. The power system has the following components:

- Two generation units Generator 1 and Generator 2 with 13.8 KVA 230 V and 5 KW and 10.3 KVA 230 V and 3 KW, respectively.
- Seven distribution lines with a typical π-models.
- Three loads each having 10 levels of parallel resistive loads ranging from 300-W to 3-kW. In this experiment, L1, L2, and L3 are set at 600 W each. L3 is considered to be a critical load, and therefore, has a redundant path to the generation units.
- Each of the five buses has three sets of three-phase inputs and outputs with 530 V/25 A solid-state relays whose switching can be controlled by digital inputs. Each phase has its own potential and current transformer for measurement data collection.

Complete specifications of the system components are found in [23,24]. It is be noted that the experimental setup is not a portion of the simulation network. It is a totally new physical system, with its own parameters and components. Contingency analysis for the experimental system were also done independently and were fed to the neural network for training. Therefore, the two neural networks, the one used for simulation and the one used for the experimental work, are different networks and are not meant to mitigate attacks for the same power network. We emphasize that the experimental setup is not a portion of the simulation network. It is rather a different small-scale testbed benchmark that was used to validate our algorithm experimentally.

### 4.2. Information Exchange and Agent Development

Figure 6c depicts the information exchange between the developed agents and the system operator at the control center, which follows the IEC 61850 GOOSE publisher/subscriber model for high speed communication. The software embedded into the agents has two threads running in parallel. The first thread is a GOOSE Subscriber. This thread listens to incoming GOOSE commands over network interface 1 and processes them through its security module before interacting with the physical power system through its digital outputs. The second thread is a GOOSE publisher. This thread waits for an internal flag from the security module to issue an alert to the system operator over the isolated and encrypted network interface.

**Figure 7:**
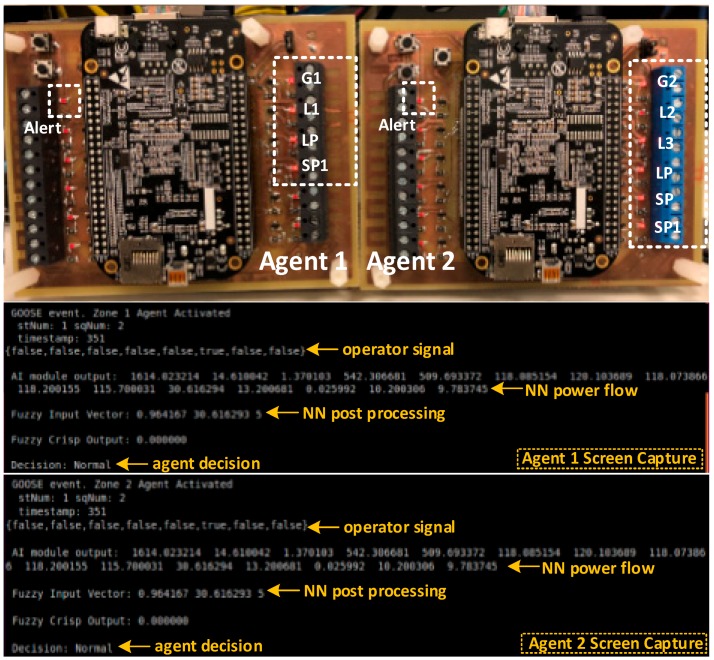
Developed agents and their corresponding human machine interface.

Figure 7 shows the actual hardware agents with their digital output extension board. The embedded microcontroller on each agent has an AM335x 1 GHz ARM^®^ Cortex-A8. 512 MB DDR3 RAM processor running a real-time Linux kernel. Agent 1 has 4 digital outputs interfaced with the circuit breakers connected to Generator 1 (G1), Load 1 (L1), the Long Path (LP) connecting busses 1 and 5, and the Short Path (SP1) connecting busses 4 and 2.

### 4.3. Results and Discussion

**Figure 8:**
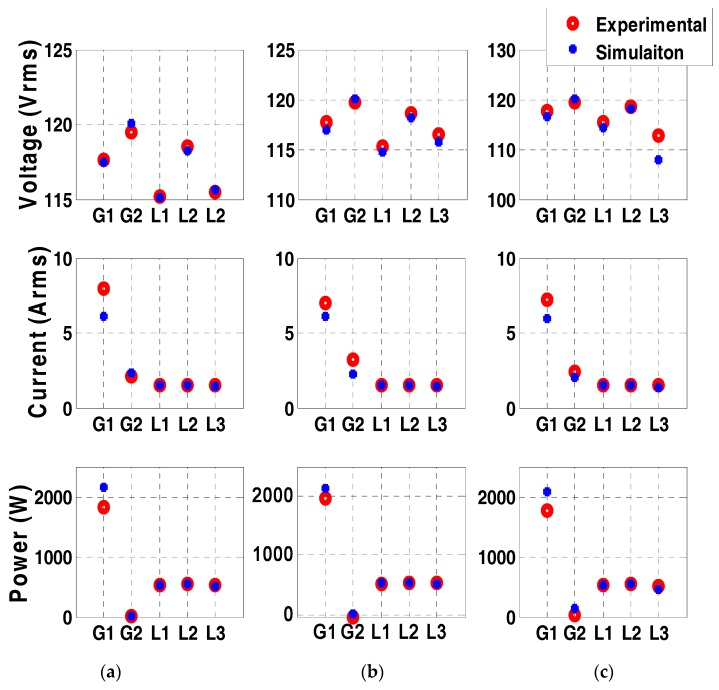
Comparison of the voltage, current, and power on all 5 buses between the simulation model and experimental setup in three cases: (**a**) normal case; (**b**) supplying CL3 from redundant path only; (**c**) under voltage case on CL3.

A data set including bus measurements of previously recorded events and measurement data collected from a simulated model of the power system for various contingency cases was used to train the AI module of the two developed agents. The simulated power system was developed in Matlab/Simulink and was verified by comparing bus voltages, currents, and power measurements with experimental data for three different cases shown in Figure 8a–c. The results show that the model accurately represents the actual system. The performed experiment comprised of nine control command signal combinations, which are:

- R1: Normal condition. All circuit breakers are closed.
- R2: Disconnection of slack generator (G1).
- R3: Disconnection of generator 2 (G2).
- R4: Disconnection of load 1 (L1).
- R5: Disconnection of critical load 3 (CL3).
- R6: Disconnection of load 2 (L2).
- R7: Disconnecting the circuit breakers between bus 4 and bus 5, which represents the main path to CL3.
- R8: Disconnecting the circuit breakers between bus 1 and bus 5, representing the redundant path to CL3.
- R9: Disconnecting the path between G1 and G2.

**Figure 9:**
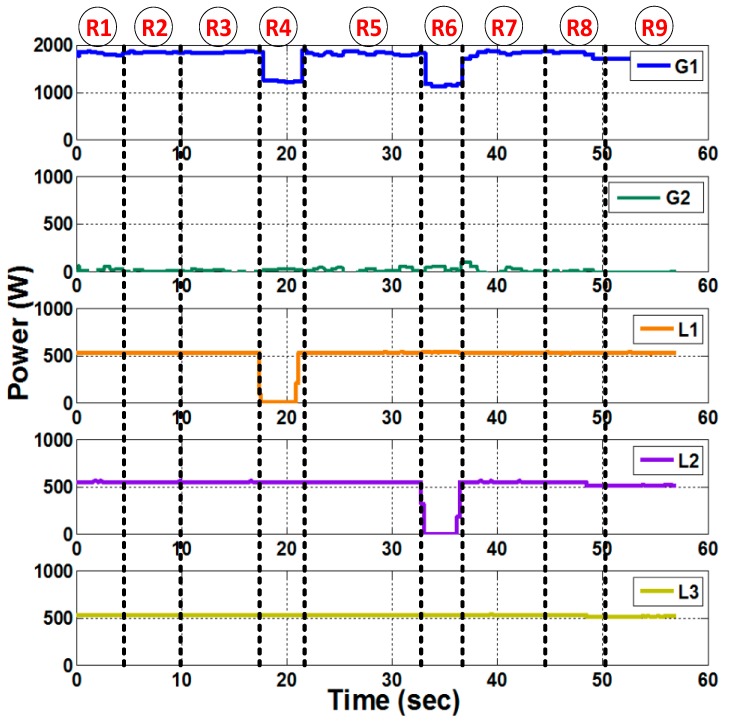
Power on each of the 5 buses of the studied power system. Each region (R1–R9) represents a different contingency case. Power drops in R4 and R6 indicate that the command to open the CB for L1 and L2, respectively, was passed by the agents.

The power measurements on each of the buses were plotted throughout the duration of the experiment in Figure 9 to visualize which control commands actually passed and which were considered as malicious, and therefore, prevented. Note that in Figure 9, there is no relation between the regions (R1–R9) and the time scale. Since this is a hardware setup, which requires careful synchronization among the generators, the power system was ran and the attacks were performed. The power was plotted throughout the duration of the experiment to show which commands were passed by the agents and which ones were blocked. Also, Table 4 shows the output of the AI module and the decision of each agent in response to each command signal. As can be seen from the results of the normal case (R1), each of the three loads was at around 600 W summing to around 1800 W, which was provided by G1. G2 was acting as a synchronous condenser. As can be seen from Table 4 and Figure 8, the commands that attempted to disconnect G1 (R2), G2 (R3), and critical load L3 (R5) were directly considered as malicious commands. This is because the override signal sent over the isolated network interface to the agents was set to zero. The disconnection of the main path to CL3 (R7) was considered a malicious command, since the AI module anticipated an under voltage of 0.90 p.u. on the critical load bus.

It is noted that the disconnection of L1 and L2 was considered as a normal command by the agents. This is shown by the drop of power on L1 and L2 from around 600 W to 0 W in Figure 9. Although this command was actually not issued by the system operator, the agents still passed these commands since they did not put the power system in an insecure state. As mentioned earlier, this is compensated for by the periodic reports that each agent sends to the system operator to take corrective action. In this experiment, after receiving periodic reports, the system operator restored power to L1 and L2, as shown in Figure 9. By the same connection, the command to open the redundant path to CL3 (8) was passed. Finally, the case where the circuit breakers connecting bus 1 to bus 2 (R9) were open, the minimum recorded voltage was 0.92 p.u. which was regarded as an alert situation and an alert signal was sent to the system operator. It is also noted in Table 4 that each agent was activated only when a change was detecting within its zone.

#### Latency Due to the Proposed Security Algorithm

A comparison of the online detection latency of the proposed method, which utilizes artificial intelligence to characterize the power system, with the work in [1], which uses a modified power flow analysis technique, is presented in this section.

The work in [1] is chosen for comparison because it targets the same type of control-related attacks detection utilizing power flow models. The results reported in [1] show that the online detection latency increases with the expansion of the system and can reach up to 200 ms. This is because their detection algorithm requires to solve the adapted power flow problem online, every time a control command is issued. Thus the time to solve the power flow problem is directly to proportional to the size of the system. However, in the proposed framework in this work, the detection latency is in 297 μs, and remains marginally constant as the number of the buses in the power system increases. This is because in this work, the computation time required for the AI module to produce an output is relatively constant regardless of the number of buses.

It is worth noting that the performed comparison is for the online detection latency. That is, detecting attacks while the system is up and running. It is true that more than 200 ms would be required to collect training data and train the AI module; however, this will be performed offline and will not significantly affect the detection latency.

## 5. Conclusions and Future Work

This paper presented a cyber-security algorithm, which utilizes artificial intelligence techniques, to defend against bad control commands targeting circuit breakers in power systems. These bad commands could be either malicious or erroneous. A multi-agent system was set up to verify the proposed algorithm. In this setup, the power system was divided into different zones and a security and control agent is assigned to each zone. Each agent hosts a trained neural network, which is able to assess the physical dynamics of the power system in response to the incoming control commands, before actually executing them. The simulation results on the IEEE 14-bus benchmark system revealed that the neural network accurately characterizes the system and is effective in blocking the power system into going to insecure states, such as having under voltages or transmission line overloading. Finally, the algorithm was experimentally verified at the FIU Smart Grid testbed, where embedded microntrollers, acting as agents, were interfaced with the physical power system over an IEC 61850-based network.

As an extension to this work, a higher level will be added the network of agents to form a hierarchy. In the upper level of the hierarchy, an agent will be present to make sure that the parameters of the neural network are adapted to accurately reflect the changes in the topology of the system, such as the addition of the new buses or power equipment. In addition to that, the neural network will be trained with a large set of *N*-k contingencies to reflect the cases where attackers launch simultaneous attacks on more than one circuit breaker.

## Figures and Tables

**Table 1:** Classification of Switching Commands and the Assumed Attacks.

Message Type	Open System Interconnect Layer	Network	Assumed Attacks
GOOSE	Layer 2 Data Link (MAC)	LAN	GOOSE Poisoning and Spoofing
R-GOOSE	Layer 3 Network (IP)	LAN/WAN	ARP Poisoning Man-in-the-Middle
DNP3.0	Layer 4 Transport (TCP/IP)	LAN/WAN	ARP Poisoning Man-in-the-Middle

**Table 2:** Ranges of Membership Functions.

		*a*	*b*	*c*	*d*
Voltage (*V* p.u.)	VL	0	0	0.872	0.92
	L	0.9	0.924	0.924	0.95
	H	0.94	1	1	1.05
	VH	1.03	1.04	2.082	2.36
*TLL* (%)	*N*	0	0	73	105
	LTE	100	115	115	130
	STE	128	148	200	200

**Table 3:** Performance of the proposed security algorithm.

					Compare w. NC			Sequential								Compare w. NC			Sequential		
	Case	V_min_ (pu)	Bus Nb.	TLL_max_ (%)	A1	A2	A3	A1	A2	A3		Case	V_min_ (pu)	Bus Nb.	TLL_max_ (%)	A1	A2	A3	A1	A2	A3
	**NC**	1.02	4	103								**NC**	1.02	4	103						
**1**	**B1–B2**	1.02	5	252							**19**	**12–13**	1.02	4	103						
**2**	**B1–B5**	1.01	5	134							**20**	**13–14**	1.02	4	106						
**3**	**B2–B3**	1.01	3	156							**21**	**GB1**	n/a	n/a	n/a						
**4**	**B2–B4**	1.01	4	180							**22**	**G B2**	n/a	n/a	n/a						
**5**	**B2–B5**	1.01	5	113							**23**	**G B3**	n/a	n/a	n/a						
**6**	**B3–B4**	1.02	5	102							**24**	**G B6**	n/a	n/a	n/a						
**7**	**B4–B5**	1.02	4	131							**25**	**G B8**	n/a	n/a	n/a						
**8**	**B4–B7**	1.02	4	130							**26**	**CL B9**	n/a	n/a	n/a						
**9**	**B4–B9**	1.02	4	123							**27**	**L B6**	1.02	4	103						
**10**	**B5–B6**	0.99	12	198							**28**	**L B5**	1.02	4	102						
**11**	**B6–11**	1.02	4	108							**29**	**CL B4**	n/a	n/a	n/a						
**12**	**B6–12**	1.02	4	103							**30**	**CL B3**	n/a	n/a	n/a						
**13**	**B6–13**	0.99	13	108							**31**	**CL B2**	n/a	n/a	n/a						
**14**	**B7–B8**	0.02	8	100							**32**	**L B14**	1.03	4	98						
**15**	**B7–B9**	1.02	14	129							**33**	**L B13**	1.02	4	103						
**16**	**B9–10**	1.02	5	108							**34**	**L B12**	1.02	4	102						
**17**	**B9–14**	0.99	14	117							**35**	**L B11**	1.02	4	102						
**18**	**10–11**	1.02	4	107							**36**	**L B10**	1.02	4	102						

NC: Normal Case; B: Bus; G: Generator; L: Load; CL: Critical Load; A1: Agent 1; A2: Agent 2; A3: Agent 3; V: Voltage; TLL: Transmission Line Loading; Yellow: Alert; Red: Malicious; Green: Normal.

**Table 4:** Experimental Evaluation of the proposed security system.

Cases (Figure 8)	Description	V_min_ (pu)	Bus Nb.	TLL_max_ (%)	A1	A2
R1	NC	0.95	3	40.8		
R2	Open CB G1	---	---	---		
R3	Open CB G2	---	---	---		
R4	Open CB L1	0.96	5	30.6		
R5	Open CB CL3	---	---	---		
R6	Open CB L2	0.96	3	30.5		
R7	Open CB B4–B5	0.90	5	40.0		
R8	Open CB B1–B5	0.95	3	40.9		
R9	Open CB B1–B2	0.92	3	52.7		

Yellow: Alert; Red: Malicious; Green: Normal.

## Appendix A. Parameters of the Simulation and Hardware Setups

Parameters of the Simulated 14-bus IEEE Benchmark System [22].

**Generator Number**	**Real Power (MW)**		**Reactive Power (MVAR)**	
	**Min**	**Max**	**Min**	**Max**
1	10	160	0	10
2	20	80	−42	50
3	20	50	23.4	40
4	--	--	-6	24
5	--	--	-6	24

**Bus Number**	**Load Real Power (MW)**	**Load Reactive Power (MVAR)**
1	0	0
2	21.7	12.7
3	94.2	19.1
4	47.8	-3.9
5	7.6	1.6
6	11.2	7.5
7	0	0
8	0	0
9	29.5	16.6
10	9.0	5.8
11	3.5	1.8
12	6.1	1.6
13	13.8	5.8
14	14.9	5.0

**Line Number**	**From Bus**	**To Bus**	**MVA Rating**
1	1	2	120
2	1	5	65
3	2	3	36
4	2	4	65
5	2	5	50
6	3	4	65
7	4	5	45
8	4	7	55
9	4	9	32
10	5	6	45
11	6	11	15
12	6	12	32
13	6	13	32
14	7	8	32
15	7	9	32
16	9	10	32
17	9	14	32
18	10	11	12
19	12	13	12
20	13	14	12

Parameters of the Hardware Setup [23,24].

**Generator Number**	**Real Power (KW)**		**Reactive Power (KVAR)**	
	**Min**	**Max**	**Min**	**Max**
1	0	5	0	4.8
2	0	3	0	7.5

**Bus Number**	**Load Real Power (W)**	**Load Reactive Power (VAR)**
1	0	0
2	0	0
3	600	0
4	600	0
5	600	0

**Line Number**	**From Bus**	**To Bus**	**KVA Rating (3-Phase)**
1	1	2	5.4
2	1	3	5.4
3	1	5	5.4
4	2	4	5.4
5	4	5	5.4
